# The Usefulness of Ultrasonography as a Guide for the Treatment of Delayed Diagnosed Tendon Injury in a 2-Year-Old Child

**DOI:** 10.1155/2013/310495

**Published:** 2013-04-21

**Authors:** Issei Nagura, Takako Kanatani, Masatoshi Sumi

**Affiliations:** Department of Orthopaedic Surgery, Kobe Rosai Hospital, 4-1-23 Kagoike-dori, Chuo-ku, Kobe 651-0053, Japan

## Abstract

In children, flexor pollicis longus (FPL) tendon injuries are uncommon. In delayed diagnosed cases, CT and MRI are hard to perform, even though to confirm the location of the lacerated proximal tendon end is preferable for the planning of operation procedure. In such condition, ultrasonography is suitable because of its characteristic feature of easy-to-perform procedure even in children. In this report, preoperative ultrasonography was practical in the delayed diagnosis of FPL tendon in a 2-year-old child to schedule the primary repair because the precise location of both FPL proximal and distal ends was identified. In addition, routine postoperative ultrasonography was also useful to track its healing process without concern about mutual communication due to the patient's age, which helped to promote active motion.

## 1. Introduction

Since FPL tendon injury is rare in children [1–3], it tends to be missed out and gives rise to delayed diagnosis. In delayed diagnosed cases, for a choice of the operation procedure of primary repair or tendon graft, it is important to evaluate the tendon condition: the location of both ends of the ruptured tendon. However, CT and MRI are hard to perform in children. Recently, ultrasonography is recognized as a practical tool for the diagnosis of the tendon injuries [4–6], and its application is expanding. This report introduced the usefulness of ultrasonography in a diagnosis, the evaluation of preoperative tendon condition, and tracking the postoperative healing process of an FPL rupture in a 2-year-old child case. 

## 2. Case Report

**Figure 1:**
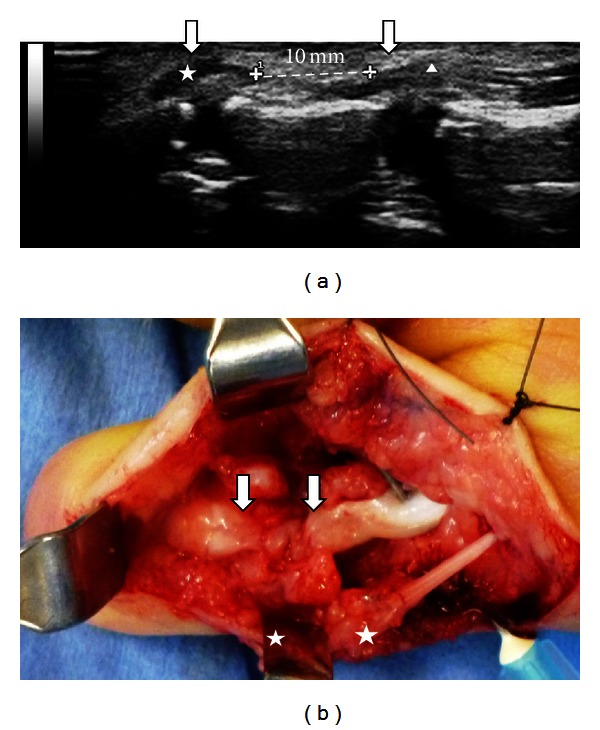
(a) Preoperative finding of the ultrasonography: the discontinuity of the FPL and both ends of the ruptured FPL tendon were shown. The asterisk showed the distal lacerated FPL tendon, and the triangle showed the proximal lacerated FPL tendon. The gap was calculated approximately 10 mm. (b) Operative findings: the primary suture was possible for the lacerated FPL tendon (arrows) and for the radial digital nerve (asterisks).

**Figure 2:**
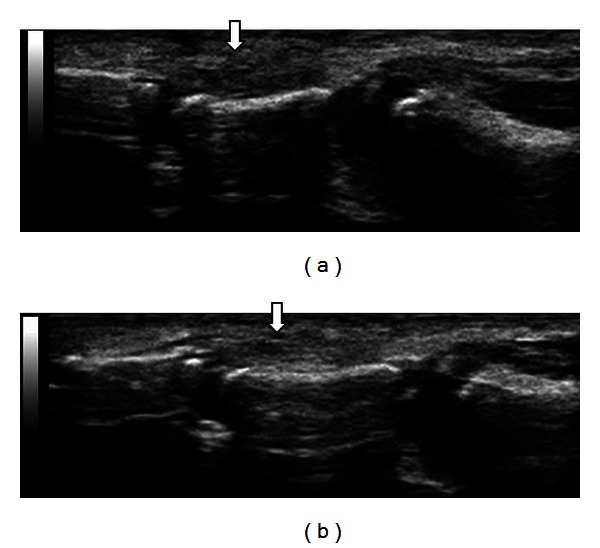
(a) Findings of the ultrasonography at three months after the surgery: it showed the edematous FPL tendon with the hypoechoic lesion. The arrow showed the suture site of the FPL tendon. (b) Findings of the ultrasonography at one year after the surgery: it showed the uniform thickness of the repaired FPL tendon. The arrow showed the suture site of the FPL tendon.

A 2-year-old child cuts his left thumb with a knife accidentally. After 2 months, his mother noticed that the active flexion of his left thumb interphalangeal joint was not possible. On his first visit to our hospital at 10 weeks after the injury, the scar of the wound was admitted on his volar side of the thumb in Zone T1. The FPL tendon laceration was diagnosed, but the sensory deficit was not clear. MRI and CT were not available for a 2-year-old child without sedation, but ultrasonography (MyLab Five, Nippon Sigmax Co., Tokyo, Japan) was able to perform easily without sedation. The ultrasonography demonstrated both ends admitted at Zone T1 level and under the A1 pulley each (Figure 1(a)) with the gap of approximately 10 mm in the sagittal section, which supported the possible direct suture. The total inspection time was approximately 5 minutes. The operative finding was equal to the preoperative ultrasonography findings with the radial digital nerve injury (Figure 1(b)). The FPL tendon was sutured directly with two strands suture using 6/0 nylon augmented by circumferential epitendinous suture, and the radial digital nerve was sutured by 11/0 nylon. Immobilization was achieved with an above elbow cast for four weeks. The postoperative course was uneventful. The ultrasonography showed the edematous FPL tendon and the hypoechoic lesion at two months postoperatively (Figure 2(a)). Additionally, adhesion of the repaired FPL tendon was shown at the proximal phalanx level in active flexion; however, those were gradually reduced showing the healing process of fibrillar pattern and disappeared at eight months postoperatively. At one year postoperatively, the active range of motion of IP joint was 0−50 degree without showing any discomfort, where the ultrasonography showed the uniform thickness of the repaired FPL tendon with a good FPL tendon excursion in the sagittal section (Figure 2(b)). 

## 3. Discussion

Although FPL tendon injury is uncommon in children, the primary FPL tendon repair leads to satisfactory results when it occurred [1, 2]. While for chronic cases by delayed diagnosis, primary repair is usually hard, and the clinical results are not satisfactory when the tendon graft was performed [3]. Therefore, in delayed diagnosed cases, the precise preoperative information about the tendon condition is crucial for the operation planning due to the differential prognosis. However, successful objective therapeutic solutions for the delayed diagnosed cases in children were not reported previously because CT and MRI are hard to perform without sedation due to their age. On the other hand, ultrasonography is well recognized as a practical tool for the diagnosis of the tendon injuries [4, 5] and relatively easy to perform in children. In our case, the patient was cooperative and the precise condition of the FPL tendon was obtained preoperatively. Then, we could plan the primary tendon repair. Moreover, ultrasonography was useful to track the postoperative healing process of the FPL tendon at consultation. There were no reports that tracked the healing process of the repaired tendon using the ultrasonography. 

The advantage of ultrasonography is that it provides real-time images instead of the static images provided by MRI [6]. Its findings were persuasive to explain the precise state of the repaired FPL tendon simultaneously and helped to promote active motion. Ultrasonography is a practical therapeutic tool to evaluate tendon condition in children who do not tolerate CT and MRI without sedation. Most tendon injuries are diagnosed clinically without ultrasonography even in children. Hence, ultrasonography could be reserved for highly selected cases, such a case is reported here, where the diagnosis is in doubt, whether in the acute setting or where the diagnosis is delayed.
